# The dementia–nature–inclusivity nexus and the needs of people living with dementia

**DOI:** 10.1017/S0144686X24000199

**Published:** 2024-11-11

**Authors:** Sally Stapley, Stephen Page, Hannah Wheat, Steve Owen, Katie Ledingham, Stephan Price, Joanne Connell, Catherine Quinn, Carol Opdebeeck, Christina Victor, Linda Clare

**Affiliations:** 1 University of Exeter Medical School, Exeter, UK; 2 Hertfordshire Business School, University of Hertfordshire, Hatfield, UK; 3 Faculty of Health: Medicine, Dentistry and Human Sciences, University of Plymouth, Plymouth, UK; 4 University of Exeter Business School, Exeter, UK; 5 Centre for Applied Dementia Studies, Bradford University, Bradford, UK; 6 Wolfson Centre for Applied Health Research, Bradford, UK; 7 Department of Psychology, Faculty of Health and Education, Manchester Metropolitan University, Manchester, UK; 8 Brunel University, Uxbridge, UK; 9 NIHR Applied Research Collaboration South-West Peninsula, Exeter, UK

**Keywords:** dementia, carer, outdoor leisure, accessibility, inclusion

## Abstract

Understanding how to improve the physical and cognitive accessibility of visitor economy businesses and organisations wanting to offer nature-based outdoor pursuits for people with dementia is key to supporting their inclusion and agency. The aim of this qualitative study was to understand the experiences, needs and preferences of people with dementia participating in nature-based outdoor pursuits in their leisure time. Semi-structured interviews were conducted with 15 people with dementia and 15 family members and subjected to thematic analysis. Four themes related to inclusion for people with dementia and their family members reflected diversity in individual needs and preferences for engaging with nature-based outdoor pursuits, their own adaptations to maintain access including accommodating risk, how cognitive and physical accessibility can be supported by businesses, and which practical and psychosocial barriers prevent inclusion. Learning from people with dementia and their family members has helped bridge the gap to their inclusion in nature-based outdoor pursuits. Their insights will inform the development of such pursuits by businesses and organisations as well as future work into risk decision-making.

## Introduction

The leisure habits of people with dementia are an emergent theme in social science research (*e.g*. Genoe, 2010; Genoe and Dupuis, 2014) as researchers recognise the leisure context within which a great deal of therapeutic interventions and activities associated with living well with dementia occur (Martyr *et al*., 2018; Bjørkløf *et al*., 2019). These issues must be set against the theoretical debates over the paradoxical nature of the ability to access leisure or engage in leisure pursuits, at the same time as potential constraints also increase (Nimrod and Shrira, 2014; Connell and Page, 2021). Although complex, leisure is non-work-related activity, vital for our wellbeing, where ‘enjoying the activities we pursue in our leisure time gives us personal enjoyment, relaxation, personal fulfilment and a sense of pleasure to make us satisfied and complete human beings’ (Page and Connell, 2010: 1). For people with dementia, the theoretical advances in understanding of how their leisure is constructed and negotiated, and unfolds in time and space, remain weakly articulated. This is because most studies are *about* people’s leisure rather than being embedded in a deeper understanding of their personal leisure lives and how they encounter, understand, create (and typically co-create with a carer or family member) experiences that have meaning to them. Keady *et al*. (2022) is perhaps one of the most interesting developments since it rethinks the emergent recognition of that deeper theoretical meaning of the experiential ‘moment’ and how that unfolds in leisure experiences. These ‘moments’ often have deep meaning for the individual and are perhaps most easily recognised in out-of-home experiences, particularly where they occur in nature-based settings. This also has a considerable resonance with the arguments around people-centred research, especially in relation to nature (Hendriks *et al*., 2016).

To date, mainstream leisure research has offered few theoretical advances in understanding the leisure–dementia nexus at a holistic level, with most studies of leisure activities unconnected with the wider leisure behaviour delivered or managed by the visitor economy. Yet the leisure literature on ageing (Page and Connell, 2022) indicates that in older age, a degree of continuity and change exists in people’s leisure. From the existing dementia literature, we may posit that the condition acts as a disruptive, adaptive or constraining influence on the leisure time of those with dementia and their carers that occurs alongside previous leisure habits and behaviour. To date, the limited evidence illustrates that leisure is still a valued and central part of the lives of people affected by the experience of dementia, as a way to add meaning and create those ‘moments’ of enjoyment, particularly in leisure undertaken out of the home. Yet in juxtaposition to this is a deeper recognition of the potentially exclusionary nature of dementia (Biggs *et al*., 2019) that poses significant challenges for policy makers and the supply of leisure resources. Meaningful activities for people with dementia are those which are significant for the individual, reflecting their individual needs and preferences, and how these are constructed in time and space. Nature and natural environments have also become an emergent paradigm in social science, as countries transition from their pandemic experiences that recognised the wider health benefits of being connected to nature, something which was already known with regard to people with dementia (Han *et al*., 2016). Bennett *et al*. (2022: 2351) state that access to ‘meaningful outdoor activities’ is a basic human right, with activities including walking, gardening and farming seen as beneficial to support wellbeing and quality of life as well as social interaction, self and identity, building upon the identification of these attributes through embodiment methodologies in Keady *et al*. (2022).

Reviews of the psychological benefits to wellbeing of nature-based outdoor activities, such as those related to horticulture, access to gardens and woodlands (*e.g*. Gibson *et al*., 2017) have been recognised in systematic reviews (*e.g*. Mmako *et al*., 2020; Murroni *et al*., 2021; Scott *et al*., 2022), with the main focus being organised activities for groups. The effectiveness of horticultural therapy on cognitive function, agitation, positive emotion and engagement for people with dementia in residential care has been reported, although further, high-quality studies are needed (Zhao *et al*., 2022). Zieris *et al*. (2023) have reported the positive impacts of bird-watching on cognitive resources, mobility and wellbeing, although again this study has methodological limitations and was with residents in nursing homes only. Collins *et al*. (2023) found that research evidence on leisure activities for older people with cognitive impairment focused on three main areas: green day care, equine-assisted interventions and community nature-based activities which included horticulture but also walking and urban woodland activity programmes. For example, Noone *et al*. (2017) have highlighted the importance of horticulture to promote physical and mental wellbeing, as well as social connectedness for people with dementia living in the community, with a community gardening project demonstrating the potential of such interventions to support agency and social citizenship (Noone and Jenkins, 2018). Yet our knowledge is largely centred on people with dementia living in or attending care facilities, with fewer studies on those living in the community, and young onset dementia is a neglected area.

Alongside organised leisure and wellbeing interventions to facilitate nature-based experiences, a broader philosophical change has emerged, extending the public policy debate on the leisure lives of people living with dementia in terms of inclusivity and human rights. This is typically framed through the social model of disability in leisure research (*e.g*. Moussouri, 2007) as an enabling paradigm where facilitating access to outdoor space is one route to leisure inclusivity because of the positive contribution it makes to supporting agency and citizenship for people with dementia (Argyle *et al*., 2017; Bartlett, 2022). This policy debate has also permeated many private and third-sector organisations which manage leisure resources and access to outdoor space in the visitor economy, as leisure inclusion is being more formally used in international policy advice on dementia (World Health Organization, 2021). However, this is not yet a cornerstone of dementia research and has had limited application to nature-based outdoor activities. The Patient and Public Involvement and Engagement (PPIE) group for this current study disliked the term ‘nature-based outdoor activities’ because ‘activities’ suggests passive engagement with activities provided *for* them rather than initiatives to support independence, agency and access; instead they preferred the term ‘nature-based outdoor pursuits’ which is the term now preferred in this paper.

Aligned with framing dementia as a disability (*e.g*. Shakespeare *et al*., 2019; Cahill, 2022), inclusion and inclusive citizenship specifically involves a focus on fairness and agency, collective action, and recognising and respecting differences (Lister, 2007), where people with dementia are not discriminated against (Bartlett and O’Connor, 2007; Bartlett, 2016). It is an implicit assumption in seeking to normalise how society, businesses and organisations perceive and treat people with hidden conditions such as dementia. Accessibility is a mainstay of this normalisation process that has evolved through the dementia-friendly communities movement (Sturge *et al*., 2021), including the broad spectrum of leisure resources that these communities support, which encompasses natural environment settings. ‘Accessibility’ refers to ensuring resources are available to as many people as possible and includes the ‘ability to access’ resources and services in relation to human needs (Disability Information Bureau, 2022). The concept of ‘cognitive accessibility’ expands this definition to refer to access that enables those ‘from a population with the widest range of cognitive characteristics and abilities to achieve a specified goal in a specified context of use’ (Steel and Janeslätt, 2017: 386). Natural England (2016) found that 55 per cent of family members stated that the person living with dementia also had a co-morbid health condition or a physical disability which affected their access to nature and outdoor spaces, reinforcing the arguments about accessibility. Few studies have considered accessibility to green spaces and barriers to inclusion for people with dementia (Mmako *et al*., 2020). Yet there is over 50 years of extant research in the leisure field that has examined these issues (Page and Connell, 2010), although not specifically related to people with dementia. However, promoting social inclusion for people with dementia is imperative and access to nature-based outdoor pursuits must consider both physical and cognitive needs in seeking to enable leisure participation.

There may also be a tension between promoting social inclusion and countering perceived risk, experienced by both leisure participants and leisure resource providers in relation to outdoor pursuits. Risk may be a key concern of family members and formal carers (Mapes, 2017; Mmako *et al*., 2020), as well as for organisations with public liability for the sites they manage. Sitting uncomfortably alongside the personhood narrative (Kitwood and Bredin, 1992; Kitwood, 1997), risk is a pervasive influence, particularly relating to residential care, and this includes perceived risks linked to going outdoors (*e.g*. Thom and Blair, 1998). Marsh and Kelly (2018: 308) report how ‘the caring landscape is firmly risk-averse’, with Argyle *et al*. (2017: 1006) arguing for ‘a proportionate balance between rights and risks’ and for collaborative working in order to develop appropriate initiatives to facilitate access to the outdoors for people with dementia. Existing research on accessibility and dementia has not specifically examined risk factors for people living with dementia but best practice guides, such as Visit England (2019), highlight the importance of site audits in designing dementia-friendly sites (Mitchell *et al*., 2003) and nature-based outdoor pursuits which balance risk with promoting inclusion.

How this inclusivity balance can be achieved may be informed by insights from people with dementia themselves. There is certainly considerable potential for co-creativity in designing such environments (Van Schaik *et al*., 2008; Zeilig *et al*., 2019). Bartlett (2022) describes the ‘access work’ performed by people with dementia wanting to engage with outdoor space such as using Global Positioning System (GPS) technologies. Such ‘access work’ is not without risk but people with dementia can be aware of their ‘vulnerabilities’ and engage active strategies to negotiate these (Bartlett and Brannelly, 2019), therein demonstrating how engagement and risk are balanced. Similarly, ‘dignity of risk’, supporting the ethical imperative of enabling risk-taking to maintain the dignity of people with dementia, is complemented by concepts such as ‘therapeutic risk’ or ‘positive risk-taking’ (Marsh and Kelly, 2018), where the potential benefits to health and well-being of taking risks are set against the negative impacts of risk avoidance (Morgan and Williamson, 2014). Positive risk-taking for people with dementia has been considered in relation to nature-based outdoor pursuits (Mmako *et al*., 2020) such as community gardening projects (Marsh *et al*., 2018) and dementia adventure holidays (Mapes, 2017), although this remains an operational issue for many organisations albeit not explicitly discussed.

Opportunities for engaging with outdoor nature-based pursuits as a normalised leisure activity are demonstrated by the importance of such visits among the general population (Office for National Statistics, 2017), and from an inclusionary perspective, should be equally available for people living with dementia who want to engage in these pursuits. However, this implies the need to make these accessible and take account of both cognitive and physical impairments, and the need to balance potential risks and benefits. As a starting point, and with the focus on inclusion and accessibility, we need to understand more about how people with dementia engage with nature-based outdoor pursuits and issues of risk in order to help understand how that accessibility can be facilitated. This study reports data from people living with dementia so that further opportunities for dementia-friendly nature-based outdoor pursuits may be developed by visitor economy businesses and organisations to expand access. The study asked a series of interconnected research questions:

- What nature-based pursuits do people living with dementia engage in?
- What are their preferences and experiences as regards nature-based pursuits?
- What gets in the way of them getting out into nature as much as they would like?

## Method

### Design

This was a qualitative, critical realist study using thematic analysis (Braun and Clarke, 2021b) to explore the accounts of people living with dementia and their family members. The critical realist stance views reality as a knowable world which exists independently of a researcher’s ideas but accepts that human practices shape how we experience, communicate and understand this reality (Maxwell, 2012). Therefore, this ontological approach is commensurate with the explanatory purpose of the study, whereby descriptive identification of experiences, needs and preferences to inform nature-based outdoor pursuit initiatives is central. Ethical approval for the research was provided by the University of Exeter Research Ethics Committee.

### Recruitment and selection of participants

Participants were selected within a UK context, which was the focus of the funded project, between November 2021 and March 2022. We identified potential participants through two specialist partner organisations and through the Join Dementia Research online portal. To be included, participants had to be either older community-dwelling individuals who identified themselves as living with dementia or cognitive impairment, or family members of such individuals who might or might not necessarily identify as ‘carers’. Participants had to speak English, be willing to be interviewed online or via telephone, and have capacity to provide informed consent. In practice, the participants with cognitive impairment were all diagnosed with dementia. Some family members were currently supporting people with dementia, while others were sharing experiences of caring for someone who was now in a care home or had since passed away. We planned to recruit and interview 30 participants, 15 people with dementia or cognitive impairment and 15 family members, chosen as a typical sample size used in qualitative studies in dementia research. However, we were open to recruiting additional participants and conducting further interviews if the resulting data did not provide sufficient depth or ‘information power’ (Malterud *et al*., 2016) to answer our research questions.

### Data collection

Interviews were conducted remotely via telephone or a video call. We used this method because, at the time of data collection, COVID-19 social distancing rules precluded face-to-face contact. The interviews typically lasted around 60 minutes. Informed consent was established prior to starting the interview. For people living with dementia, we gained informed consent verbally over the telephone or video call. People living with dementia and family members were sent a ‘welcome pack’ which provided information about the study, a participant information sheet and an online consent form, which they completed before the scheduled call. With the exception of one mother–daughter dyad, interviewees were unknown to each other, therefore no joint interviews were conducted.

The interview schedules (*see* the online supplementary material) were developed with the involvement of the project advisory group of people with dementia and family members. These covered what nature-based outdoor pursuits people living with dementia engage in, what their preferences and experiences are, and what gets in the way of them getting out into nature as much as they would like. The semi-structured format encouraged rich data collection by giving researchers opportunities to ask exploratory questions about individuals’ experiences and perceptions, and to explore new avenues of interest, allowing the interaction to steer the trajectory of the interview (DeJonckheere and Vaughn, 2019). Photographs of various outdoor scenes and pursuits were emailed to all telephone and online interviewees prior to the interview. These photographs were used to stimulate discussion by asking whether the given pursuit was of interest to them and why. Photographs included images of pursuits such as walking, gardening, cycling and watching wildlife. In addition, interviewees were able to discuss their own nature-based outdoor pursuits during the interview. Interviewees were also asked to use a five-point ‘smiley face’ Likert scale to score if they were able to get outside as much as they wanted to, again to stimulate discussion. All interviews were transcribed verbatim by a professional transcription services company, subject to their signing of a confidentiality agreement.

### Data analysis

Reflexive thematic analysis was used to analyse the data, so that coding could be as ‘unstructured and organic’ as possible (Braun and Clarke, 2021*a*) and meaningful patterns identified across the qualitative dataset. We followed the six phases of reflexive thematic analysis outlined by Braun and Clarke (2021*a*): familiarisation, coding, generating initial themes, reviewing and developing themes, refining, defining and naming themes, and writing up. To begin with, SS (the main analyst) and HW (co-analyst) familiarised themselves with the dataset by reading the interview transcripts, making initial notes on their observations, and meeting to discuss these observations. This informed the initial coding by SS, which involved creating codes by tagging data with a relevant meaning to the research questions with a code that ‘evoked the coded data’s meaning’ (Braun and Clarke, 2021*b*: 236). This approach to coding necessitated having an evolving (data-driven) coding framework (managed within NVivo 2020), which increasingly reflected the interpreted meaning(s) within the overall dataset as more data were coded and understanding evolved. When all the data had been coded once, SS and HW met to revise the coding framework, based on their enhanced understanding of the data. Codes varied in terms of how much semantic and latent meaning they provided, with latent codes capturing more of the researcher’s analytic perspective on the meanings held by the data.

Initial themes were generated through the interpretation by SS of the patterns observed across the coded dataset. This involved grouping codes which seemed to share a core idea or concept and which provided insights relevant to the research question, and developing a candidate theme description or label that represented the overall meaning of each grouped set of data. Themes were reviewed and subsequently developed, named and defined by SS and HW, using the full coded dataset to determine whether the candidate themes made sense, told a separate story and best represented the most salient meanings offered by the data. The authors met to discuss their thoughts and undertake a process in which some data were assigned to different themes, and themes themselves were amalgamated, split apart and redefined. Part of the process of redefining or renaming the themes involved creating a summary of the story told by each theme. Writing up was done using PPIE feedback, individual researchers’ notes and theme summaries, alongside extracts from the data, to help share the overall picture painted by the participants’ accounts. Resulting themes were predominantly of a practical, descriptive nature due to the focus and aim of the study.

Reflexivity was considered at personal and epistemological levels (Lazard and McAvoy, 2020) throughout, by researchers attending to how data collection, analysis and other methodological decisions were influenced by personal characteristics, knowledge and experiences. For example, SS is a former carer of a relative with dementia but remained cognisant of the potential for this prior experience to impact the analysis. This process involved making short notes about how the interview process may have influenced what insights were shared, keeping records of thoughts and feelings experienced during coding, and reflecting on these when working together on the analysis. The emerging themes and narrative account were discussed with the project PPIE advisory group on 17 May 2023, with positive feedback received, and with themes resonating with PPIE members’ perspectives and experiences. These were also discussed with the co-authors to broaden the discussion about the significance of the themes in relation to the extant literature on the outdoors and dementia.

### Findings

Fifteen people with dementia and 15 family members of people with dementia were interviewed for the study. Interviewees with dementia ranged in age from 52 to 91, and family members from 55 to 90. Of those with dementia, ten were male and five female. Five male and ten female family members were interviewed, including six family members whose relative was deceased at the time of interview (*see* Tables 1 and 2). It was not possible to determine the type of dementia or severity of cognitive impairment of the person with dementia being interviewed or being represented in a family member interview. However, interview accounts reflected a range of dementia severity and included the experiences of people with young onset dementia, where symptoms first occur under the age of 65. Both people with dementia and family member interviewees also had a range of leisure-related needs related to physical as well as cognitive impairment.

Facilitating inclusion for people with dementia and their family members to engage with nature-based outdoor pursuits was the focus of the analysis, the aim of which was to provide practical guidance on how to achieve the beneficial outcomes associated with nature/outdoor pursuits which previous literature has identified (Mmako *et al*., 2020; Murroni *et al*., 2021; Scott *et al*., 2022). Our interviewees reported various perceived benefits of engaging with nature. Most interviewees discussed benefits to their mental wellbeing such as improved mood, ‘the feeling of freedom’ (Sarah) or being ‘calm and peaceful and gently happy’ (Ava). A few interviewees felt such engagement prevented decline: ‘I think that degree of stimulation and happiness maybe just kept the dementia at bay a little bit longer than would perhaps otherwise have been the case’ (Wyatt). Interviewees also discussed the benefits of getting outdoors into nature for developing new or existing social connections: ‘just being with people and company’ (Henry). Fewer interviewees emphasised the benefits or challenges of nature-based outdoor pursuits for physical wellbeing or fitness.

Interview accounts reflected a large range of activity preferences (*see* Table 1). Common choices for outdoor pursuits were going for walks in nature, looking at country or coastal views, gardening, and visiting gardens or parks. One family member contrasted her father’s experience of going to a day centre where there were no outdoor activities with what she felt he may have favoured instead: ‘I think dad would have probably preferred as much to go and sit by the lake or go for a walk along a towpath somewhere’ (Sophia). A few interviewees, including those with young onset dementia, preferred more physically active pursuits such as long walks, running or cycling.

Four themes were derived from the analysis. The first refers to the diversity of individual needs related to physical and cognitive difficulties in engaging in leisure and a range of preferences for engaging with nature-based outdoor pursuits, such as whether they were willing to take part in a group or not. The second identifies the diverse adaptations made by people with dementia and their family members to maintain access to nature-based outdoor pursuits (*i.e*. continuity in their leisure lives), as well as how they accommodate risk as dementia develops and progresses. The third considers how businesses and organisations can improve physical and cognitive accessibility for people with dementia as well as raising awareness of the condition, a feature which businesses commonly report (Connell *et al*., 2017). The final theme relates to practical barriers to inclusion which businesses and organisations can address (Visit England, 2019), as well as psychosocial barriers faced by those affected by dementia, such as lack of self-confidence or self-motivation in the person with dementia, and availability of the family member to offer support with engagement.

### Individual needs and preferences for engagement: ‘it’s not one size fits all’

This first theme concerns individual needs related to physical mobility and cognitive impairment as well as individual preferences for how interviewees wanted to engage with nature-based outdoor pursuits. One interviewee cautioned against ‘homogenising’ people with dementia, emphasising diversity in their needs and preferences:

What I would like to see people do is to also go on a dementia friends^1^ training to learn a little bit about what makes us tick. But come away from it knowing that we’re all different. It’s not ‘one size fits all’ by any stretch of the imagination. (Samantha)

Both people with dementia and family members had a range of different health problems, with physical difficulties often perceived as more problematic than those related to dementia, although distinguishing issues such as impaired balance and depth perception from dementia is complex. Physical and sensory difficulties included: hearing impairment, hyperacusis, sight issues, incontinence, arthritis, heart problems, stroke sequelae and walking difficulties. Several interviewees including family members used walking aids, and some family members reported their relatives were wheelchair users; few of the respondents had no mobility issues. Issues related to cognitive impairment and dementia included speech difficulties, becoming non-verbal in advanced dementia, sequencing difficulties affecting daily activities (*e.g*. how to make a cup of coffee), disliking crowded places and forgetting words: ‘I haven’t got lost memory, I’ve got misplaced memory’ (Peter). Therefore, people with dementia may have a range of individual needs related not only to their dementia but to other health problems which may affect their engagement with nature-based outdoor pursuits and pose specific barriers to leisure participation that change through time as the condition deteriorates (Innes *et al*., 2016).

Individual preferences for engagement varied regarding both the kinds of pursuits people wanted to do and how these were accessed. Affected by self-perceptions of ageing and ability, some individuals with dementia wanted to try new activities, continue with current ones or resume past activities, illustrating the importance of past leisure lives and a desire to maintain the enjoyment which nature offers. One interviewee had enjoyed hillwalking but had stopped due to his balance issues (Tom), whereas another no longer went running due to arthritis (Paul). A few interviewees, especially those with young onset dementia, wanted challenging pursuits. One disliked the dementia-friendly activities he had tried because ‘they’re just a bit too sedentary for me because I’m active’ (Kelvin), therein illustrating the heterogeneity in leisure needs. For one interviewee, giving up lifelong activities he had previously enjoyed was a coping strategy to manage the psychological impacts of dementia and, although keen to try fishing again, he no longer felt he could cook his catch: ‘so that probably means it would be impossible to recreate what I used to have’ (David).

The implications for inclusion are varied as some respondents wanted to go alone and be independent whereas others wanted to attend with their family members, in order to have a day out together or because they needed their support, or a combination of the two. Although some interviewees said they would be more likely to go if a business was advertised as ‘dementia-friendly’, others might not: ‘maybe not everybody wants to be hit in the face by the fact it’s dementia-friendly’ (Amelia). There were also different preferences regarding whether or not people with dementia wanted to be part of a group specifically for people with dementia, with one family member saying her father would not have joined a dementia group because he did not like admitting he had the condition: ‘so you’d have to find ways of engaging them without mentioning what it is’ (Ava). Conversely, other interviewees said they were happy to be included in groups for people with dementia or indeed other kinds of groups: ‘I quite enjoy those with the dementia focus. And I also belong to groups that don’t have a dementia focus’ (Caroline). Therefore, people with dementia are not a homogenous group but have a variety of different needs and preferences related to both physical and cognitive impairment, the kinds of activities they want to engage in, how they engage and with whom. Consequently, when designing sites (*see* Day *et al*., 2000) or businesses’ inclusion strategies, organisations need to understand individual needs and preferences of people with dementia as the long-standing literature on leisure advocates (Innes *et al*., 2016).

### Strategies for maintaining access: adaptations and risk

This second theme focuses on the diverse adaptations people with dementia and their family members make, or the work they do, to gain access to nature-based outdoor pursuits, and their largely pragmatic approaches to considerations of risk. People with dementia and their family members made adaptations in various ways, some of which might seem restrictive but were spoken about predominantly as active ways to maintain engagement with the outdoors to maintain their outdoor leisure lives. Interviewees discussed sticking to familiar walking, cycling or running routes, especially when they were on their own, and only going further or to new places when they could do this in the company of others: ‘I don’t ever try anything new on my own because of the fear of getting lost, so it’s usually familiar routes’ (Robert). Some interviewees walked less far and had more rests than they used to. One interviewee said he made sure to orientate himself before setting out on a walk: ‘I get my bearings first before I go anywhere, you know what I mean, so I know how to get back’ (John). Another had his ‘comfort zones’ walks he knew well but also pushed himself to walk further: ‘I’ll go and sort of scout it out, but I’ll always make sure I’m sticking to straight lines’ (Peter).

Interviewees avoided walks where they knew there were obvious obstacles such as stiles to negotiate, and some opted to go to areas where they knew there was flat terrain or that were wheelchair-accessible. Some family member interviewees discussed assessing the accessibility of a site before bringing their relative, ‘but you can class that from a completely disabled perspective, not necessarily just from a dementia-friendly perspective’ (Amelia), thus demonstrating the importance of leisure audits of sites for accessibility. Family members also spoke about calling venues in advance to see if they could meet the specific needs of their relatives. Some people with dementia used technology, such as keeping a mobile phone with them when they were on their own, sometimes at the insistence of a family member. Others also used GPS on a phone, used a watch for orientation, or carried an identity card with their diagnosis and a relative’s contact details, acknowledging the underlying concern with leisure risk behaviour which had surfaced as dementia progressed.

Notably, risk was not explicitly discussed in most interviews although concerns about risk were evident, *e.g*. in terms of the adaptations made by people with dementia. However, in a very few family member interviews, concerns about risk were more directly apparent. One family member who cared for both her husband and mother with dementia was concerned about her husband falling over, which meant she did not enjoy the experience of being outdoors herself: ‘from a carer’s point of view, all you’re doing is gauging how you’re walking without looking where you are, so it’s not a dual experience’ (Amelia). Another family member when asked whether her partner was worried about going out by himself responded: ‘No, not at all. That’s why I worry’ (Hazel). However, in keeping with the adaptive strategies discussed in many interviews, perspectives on risk, where evident, were largely pragmatic rather than fearful, and this included those of family members:

I had a very simple philosophy that barriers and obstacles were simply inconveniences which you had to find a way around. (Wyatt)

I’d be honest, there has been people has told me, like, you shouldn’t be going to these places. Because of me, with my balance and my vision, people have told me – ‘look, don’t be going to the towpaths! What if your balance goes and you fall in?’ And I keep saying, ‘but what if I was crossing the road and get knocked down by a car?’ We’ve all got to have risks, we’ve all got to take risks … that’s like saying to somebody who hasn’t even got dementia, ‘no, you can’t go out because what if you get knocked down crossing the road?’ I mean, we’ve all got to have some sort of risk. I mean, I’m not saying go out and (laughs), I’ve taken it seriously. You’ve got to be logical about stuff. (Peter)

Therefore, both people with dementia and their family members were able to get out and enjoy nature-based outdoor pursuits, but by making adaptations themselves rather than enjoying inclusive access. Concerns about risk were referred to in some interviews but these did not prevent engagement.

### Supporting inclusion: physical and cognitive accessibility and dementia awareness

This third theme suggests how businesses and organisations can bridge the gap to inclusiveness (Bartlett, 2022). This relates to how experiences can be devised or improved by considering physical and cognitive accessibility as well as improved understanding of dementia, typically through service blueprinting using people with dementia to help make sites accessible (Connell and Page, 2019). When asked about these issues, interviewees discussed multiple factors including wheelchair access, flat walkways and even terrains, maintaining footpaths, the visibility of steps (*e.g*. for those with depth perception problems) and having additional seating at an appropriate height. On cognitive accessibility, interviewees discussed signage for getting to and navigating the site including maps (with use of GPS also requiring good internet access), information about exhibits such as plants, and having both entrance and exit signs for toilets. The readability of signs was important such as not including too much information, particularly for the interviewee for whom English was not his first language and who felt he was losing these language skills (Robert), although this interviewee did not discuss wanting signs in other languages. In addition, staff support and availability on site was seen by some as central.

There was discussion of improving organisational and public understanding of dementia. Although no interviewees reported experiences of stigma due to dementia, most felt it was not understood by the general public or most businesses and organisations. Several interviewees discussed a lack of recognition of hidden disabilities:

I think a lot more could be done along, around the hidden disabilities, if you like. They’re all over it if they can see you’re in a wheelchair or there’s something they can see wrong with you … I mean, I’ve been to places, and I’ve worn the lanyard to show that I’ve got a disability. They don’t even have a clue what it means! (Paul)

A family member suggested that young onset dementia is not accounted for either, with the perception that dementia only affects older people:

I think if they know anything at all about dementia, it’s still something that older people get. You wouldn’t expect, you know, a physically fit 53-year-old to be suffering from dementia. No, I think the general awareness is still sadly low. We’re still in an education phase here. (Wyatt)

Interviewees reported a limited number of discriminatory responses to risk and safety where people with dementia have been excluded from accessing pursuits such as walking groups ‘because of the risk’. However, as discussed within the previous two themes, people with dementia are individuals with a range of different needs but also abilities who reconcile risk by negotiating numerous adaptive strategies for engagement. Therefore, exclusion on the basis of diagnosis alone, although thankfully not dominant across our interview accounts, is still an underlying issue in terms of public awareness, given the growing prevalence of dementia in society.

### Preventing inclusion: practical and psychosocial barriers

The previous themes have highlighted what people with dementia and their family members need to facilitate inclusion and what they do themselves to gain access to nature-based outdoor pursuits. This final theme focuses on practical considerations affecting inclusion as well as key psychosocial barriers to engagement, building on the theoretical discussion of barriers to leisure by Innes *et al*. (2016) and the social model of disability in understanding how to overcome barriers. Providing information on the availability of nature-based outdoor pursuits for people with dementia was an obvious concern: ‘well, I think the first thing is to promote themselves, because if we don’t know it’s there, then we’re never going to find out, you know’ (Kelvin). Following promotion, visitors needed to determine whether sites and their pursuits were accessible and able to meet the needs of the person with dementia:

I think perhaps on sites when you’re looking it up they could be more explicit on what it is like and whether they’re dementia friendly, whether they’re disability accessible. (Paul)

Interviewees also referred to the importance of available amenities including sufficient accessible toilets in close proximity during a leisure visit (including provision for the family member to help manage their relative’s incontinence), a quiet café, especially for those with a hearing impairment, parking, and ease of transport, particularly for people who no longer drive. The cost of getting to and engaging with the pursuit was a concern for some more than others. However, the impact of the weather was mentioned frequently, reflecting the seasonality of visitor attraction visitation among the general population (Connell *et al*., 2015). Participants disliked going out in the rain, where wheelchair-users in particular could easily become cold, ‘there was a bit of rain, and it was go, go, go’ (Amelia), also disliking windy weather ‘because it could knock us off our feet’ (Olivia). One interviewee, however, offered a practical solution to the British weather: ‘there’s no such thing as bad weather. It’s about having the right gear for it’ (Samantha).

There were psychosocial and other barriers to inclusion which may be more problematic to address. The availability of the family member to attend an organised activity or pursuit at a set time with the person with dementia was limited if the family member was employed. Yet, as with several of our interviewees, the person with dementia might want or need a family member to go with them or be required to do so by the activity organisers. One family member said her partner had been prevented from staying with the gardening group he had got to know because, when the group moved to a different garden, a family member was now required to attend. A few family members themselves wanted support while on site, such as being able to have staff stay with their relative while the family member used the toilet.

Importantly, some family member accounts emphasised psychological barriers to attending, perhaps due to dementia itself, where their relatives lacked self-confidence or self-motivation to the extent that they did not want to leave home at all: ‘he says he feels secure staying here, it’s almost like a cotton wool cocoon. It’s a safety net, isn’t it?’ (Scarlett). Consequently, some family members were not able to get out into nature themselves due to the caring role, *i.e*. where the person with dementia did not want to go out but could not be left on their own. This reticence is difficult but important to address, so that all people with dementia and their family members have the opportunity to gain the benefits which engaging with nature and nature-based outdoor pursuits can afford.

## Discussion

The purpose of this qualitative study was to understand the experiences, needs and preferences of people with dementia and their family members with regard to participating in nature-based outdoor pursuits, building on existing theoretical studies of leisure and dementia which examined barriers to participation (*e.g*. Innes *et al*., 2016), with a focus on the outdoors and nature. Broadly consistent with the findings of long-standing theoretical research on leisure constraints from social psychology (Crawford and Godbey, 1987; Godbey *et al*., 2010), we have identified what people enjoyed doing and what prevented them getting out into nature as much as they wanted to. Although our study sample is UK-based, our findings add to this theoretical corpus and are therefore likely to transfer to people living with dementia and their family members in other countries. Our account is centred on issues related to inclusion for people with dementia and their family members, something that is now a central feature of the dementia–leisure–inclusion nexus as the debate has moved on from identifying barriers to focusing on how they need to be removed in the inclusion paradigm. We have highlighted the broad spectrum of factors that shape the individual nature of that personal nexus, *e.g*. cognitive and physical health difficulties, including mobility issues, experienced by people with dementia affected engagement or continued engagement with nature-based outdoor pursuits.

In doing so, we have shown the diversity in individual preferences for engagement, such as wanting to attend independently or in a group for people with dementia. This reinforces the individuality and agency associated with responses to dementia and leisure that Genoe (2010) reported. The diverse engagement strategies adopted to continue to maintain access build on over 30 years of leisure research focused on how to negotiate and address barriers to leisure, pioneered by Jackson *et al*. (1993) in the absence of inclusionary models of access. This study illustrates that people living with dementia follow very similar approaches to barrier negotiation to maintain leisure lives through their journey with dementia. This extends the existing theoretical debates which suggest that marginalised groups are excluded from leisure access. Our study illustrates that, whilst leisure is an individualised experience, people with dementia do exhibit a range of engagement strategies and so active out-of-home leisure does not cease at the point of diagnosis. To further support the aim of inclusion, our research demonstrates the need for improvements to physical and cognitive accessibility, where, for example, accommodating people with different levels of cognitive impairment is needed to ensure equitable access. There is also a case for raising awareness of dementia as a hidden disability, particularly young onset dementia, recognising an underlying tension surrounding risk.

Practical barriers to inclusion such as lack of information on accessibility were reported, as well as psychosocial barriers such as the person with dementia lacking self-motivation that are well known in the leisure literature. Consequently, our study demonstrates a contribution to knowledge on accessibility and inclusion underpinned by leisure theory. Specifically, it illustrates how people negotiate barriers and personally facilitate experiences that are not organised visits. This starts to build a picture of the way in which some people with dementia demonstrate resistance to the condition, seeking to live well and not let the condition destroy their leisure lives (Genoe, 2010). In contrast, for others resistance is replaced by anxiety and aversion to the outdoors and nature, and so for these individuals, some degree of intervention may be helpful to stimulate and encourage participation to rekindle their confidence and overcome negative perceptions of what they can and cannot do. Above all, our study reinforces the argument that the leisure lives of people with dementia deserve much greater attention. Concepts of normalisation and inclusion are not sufficient to illuminate what shapes their outdoor leisure lives and behaviour. These highly personalised leisure lives need to be understood in order to derive broader generalisations for leveraging nature experiences. This will help people live well with dementia so they do not feel excluded from society, if they have a desire to continue accessing nature. It also illustrates that there is a considerable way to go in closing the gap between expectations and needs for nature visits and specific access requirements.

For businesses and organisations, the range of physical and cognitive health needs of people with dementia mean that cognitive accessibility (Steel and Janeslätt, 2017) and access in relation to physical health needs emerge as key drivers for future changes in site development. Visits to leisure sites for people with dementia may involve many permutations of the type of engagement designed, including how family members or supporters of the person with dementia are involved, and whether it is a shared experience for them both. In addition, because people with dementia may not necessarily be prepared to attend dementia-specific groups and may also avoid anything labelled as relating to dementia, there needs to be consideration of how access for people with dementia can be improved more generally across the business or organisation rather than limiting this to specific forms of provision only.

Practical solutions to support inclusion will vary by business or organisation but should include provision of suitable amenities, *e.g*. adequate parking, accessible toilets and ideally quiet areas, as such factors also impact the experience of the person with dementia and their family members. Clear information on access and availability will also necessitate effective promotion of any nature-based outdoor pursuits that businesses and organisations develop. None of these issues are difficult to address if an accessibility guide is available and the site has been audited for accessibility. Among the greatest concern expressed was physical mobility, although the concerns of younger interviewees who wanted more physically challenging outdoor pursuits should not be negated. Needing wheelchair access and flat terrains will resonate with accessibility more broadly for many groups but orientation and needing signage with clear legibility as well as good internet access for GPS use is emerging as a basic hygiene factor for commercial visitor attractions that have a nature component. Staff and volunteer training in dementia, including young onset dementia and in recognising dementia as one of a range of a ‘hidden disabilities’, is certainly an emergent theme for the business sector and visitor economy (Connell *et al*., 2017). Raising awareness may also counter the discriminatory practice of excluding people with dementia from accessing nature-based outdoor pursuits ‘because of the risk’.

In line with what Bartlett (2022) reported, our interviewees were adept at making their own adaptations to maintain access to the outdoors, and to nature and nature-based outdoor pursuits specifically, although businesses and organisations learning from such adaptations and making their own changes to support access should help mitigate this need. Understanding the leisure behaviour expressed by participants, such as sticking to familiar walking routes, demonstrates active agency by people with dementia, as Ward *et al*. (2022) also observed. This is important to maintain engagement with the outdoors and seek to offset the ‘shrinking world’ often experienced by people with their journey with dementia in their leisure lives, as that world closes in on them and confines their leisure to the home environment (Duggan *et al*., 2008; Connell and Page, 2021). However, there are inherent tensions in the leisure–dementia–nature nexus where the psychosocial barriers of lack of self-confidence and self-motivation to go out may mark the final time–space compression of the ‘shrinking world’ or a more anxiety-led self-preservation *modus operandi* perhaps due to personal changes in mobility and negative attitudes towards risk. As services and interventions for managing social withdrawal in dementia are limited (Chang *et al*., 2021), how such individuals can be supported is problematic, even though they may be missing out on the well-being benefits that engaging with nature and nature-based outdoor pursuits can offer. In addition, consideration of risk was evident in the accounts of both people with dementia and their family members. Although this did not dominate our interviews, risk was a key discussion point with our PPIE group, when relating the emerging themes to their own experiences. Although it is unclear why discussion of risk was not pervasive, it may be, as our findings suggest, that, to prevent their exclusion from outdoor leisure, people with dementia are pragmatic about risk and make adaptations accordingly. Future work specifically on accommodating leisure risk, building on the leisure theory literature on risk (*e.g*. Cheron and Ritchie, 1982; Rojek, 2003) and changing behaviour among carers (Dunn and Strain, 2001), would help to understand who develops adaptive strategies towards leisure in the outdoors and how they substitute, modify and potentially cease certain forms of leisure in nature.

Our study is not without its limitations. At the start of recruitment and interviewing, researchers became aware that the first interviews were generated via project partners’ networks, and so involved people who may be considered ‘dementia advocates’ and hence perhaps not representative of the majority. This may also be why risk did not dominate the interviews, although equally the assumption that it would may be erroneous. Within a qualitative sample, our subsequent broadening of recruitment to redress this may not have fully countered this issue, potentially overlooking everyday leisure experiences of people living with dementia and their family members. Conversely, the inclusion of ‘dementia advocates’ within the sample may have afforded useful insights into the topic and does not appear to have biased the findings, given that these are broadly consistent with many of the issues raised by Innes *et al*. (2016). We are also aware of the largely White British demographic of our interviewees, and further work with a more ethnically diverse sample of participants may be needed as advocated elsewhere (Low *et al*., 2019). In addition, family members as ‘carers’ of someone with dementia were not given focus as to their own experiences, needs and preferences for engaging with nature-based outdoor activities.

Nonetheless, this study is one of very few to have explored how people with dementia living in the community engage with nature and nature-based outdoor pursuits independently in their leisure time, identifying diversity in individual needs and preferences for engagement as well as demonstrating the adaptations they make, the challenges they face, and how businesses and organisations can better facilitate inclusion. The study is encouraging because it has a degree of commonality with many of the typical issues raised in the context of an ageing demographic, reflecting leisure behaviour trends among an ageing population and the challenge for businesses and organisations in catering for more heterogeneous leisure needs. The study has much greater international significance as the scale and challenge of dementia continues to grow and contributes to highlighting the changes that can be made to normalise the experience of nature through better access for all. By understanding how to accommodate and maintain the leisure lives of people on a journey with dementia, we start to see a degree of continuity and change in the way they engage with nature or how we may need to motivate others to engage with nature to stay active and to live well with dementia. This will continue to be a key priority to help maintain community-based living whilst offering an opportunity for the managers of nature sites and pursuits to embrace greater diversity and creativity in their visitor markets.

## Supplementary Material

The supplementary material for this article can be found at https://doi.org/10.1017/S0144686X24000199.

1

2

## Figures and Tables

**Table 1:** Person with dementia: sociodemographic characteristics and nature-based outdoor pursuits preferences

Participant: person with dementia^1^	Age	Gender	Ethnic background	Living arrangements	Family member interviewed?	Nature-based outhoor pursuit preferences
Tom	70	Male	White British	With wife	No	Walking on the beach; bird watching; walking dog; local circular walk
Paul	67	Male	White British	With wife	No	Metal detecting; fishing; walk around local park; bee keeping with son; managing small back garden; walking around garden estates; foraging; travelling abroad
Henry	52	Male	British (ethnicity not stated)	With partner	No	Walking in woods; going for long walks; open water swimming; running; cycling
David	Not reported	Male	White British	Alone	No	Visiting castle grounds; being near water; visiting local park with lake; Sport England walks; walking; travelling in UK
Kelvin	70	Male	White British	Alone	No	Sailing; fishing; camping; walking; National Trust site; coast; foraging; travelling UK/abroad
Sarah	70	Female	White British	With husband	No	Walking around lanes with daughter; travelling UK/abroad
Mathew	90	Male	White British	Alone	No	Circular walks in countryside; walking group; gardening; travelling UK with family; looking at views
John	66		White British	With wife	No	Walking dog; walking group; gardening with men’s group
Peter	62	Male	White British	Alone	No	Walking; walking groups; interacting with animals; travelling local region
Caroline	76	Female	White British	With husband	No	Walking in woods; cycling; foraging; visiting gardens; outside group (various activities); travelling UK/abroad
Lucy	91	Female	British (ethnicity not stated)	Alone	No	Trips with University of the Third Age; looking at views; walking local parks; watching wildlife
Samantha	66	Female	White British	With partner	No	Running; walking around local fields; walking; visiting Brecon Beacons; travelling UK/abroad
Robert	Over 60	Male	Middle Eastern	With wife	No	Walking dog; travelling UK/abroad; foraging
Mia	Not reported	Female	British (ethnicity not stated)	Alone	Yes (Nora)	Walking dog in the forest; walking dog in local fields; seeing/hearing wildlife
James	70	Male	White British	With wife	No	Playing golf; walking around woods; fishing; walking around cemetery; gardening

1 All names are pseudonyms. UK: United Kingdom.

**Table 2:** Family member: sociodemographic characteristics and relationship with the person with dementia

Participant: Family member^1^	Age	Gender	Ethnic background	Lives with person with dementia?	Relationship with person with dementia
Olivia	75	Female	British (ethnicity not stated)	Yes	Wife
Noah	72	Male	Not reported	Yes	Husband
Emma	64	Female	British (ethnicity not stated)	No	Daughter
Charlotte	57	Female	White British	No	Daughter
William	76	Male	Not reported	No	Husband (wife deceased)
Amelia	70	Female	White British	Yes (with husband)	Wife (mother also has dementia)
Lucas	58	Male	White British	No	Son (mother deceased)
Ava	69	Female	Not reported	No	Daughter (father deceased)
Wyatt	69	Male	White British	No (wife living in a care home)	Husband
Sophia	60	Female	White British	No	Daughter (both parents had dementia – both deceased)
Luke	90	Male	Not reported	No	Husband (wife deceased)
Isabella	55	Female	Black British	No	Daughter (father deceased)
Scarlett	70	Female	White British	Yes	Wife
Nora	56	Female	White British	No	Daughter
Hazel	62	Female	White South American/British	Yes	Partner

1 All names are pseudonyms.

## Note

1 The interviewee is referring to dementia friends training devised by the Alzheimer’s Society in the UK to change people’s perceptions about dementia.

## Data

Qualitative data will be deposited with ESDS Qualidata within 3 months of study completion. On project completion, selected data will be deposited with the University’s Institutional Repository, Open Research Exeter (ORE).

## References

[R1] Argyle E, Dening T, Bartlett P (2017). Space, the final frontier: outdoor access for people living with dementia. Aging & Mental Health.

[R2] Bartlett R (2016). Scanning the conceptual horizons of citizenship. Dementia.

[R3] Bartlett R (2022). Inclusive (social) citizenship and persons with dementia. Disability & Society.

[R4] Bartlett R, Brannelly T (2019). On being outdoors: how people with dementia experience and deal with vulnerabilities. Social Science & Medicine.

[R5] Bartlett R, O’Connor D (2007). From personhood to citizenship: broadening the lens for dementia practice and research. Journal of Aging Studies.

[R6] Bennett J, Wolverson E, Price E (2022). Me, myself, and nature: living with dementia and connecting with the natural world – more than a breath of fresh air? A literature review. Dementia.

[R7] Biggs S, Carr A, Haapala I (2019). Dementia as a source of social disadvantage and exclusion. Australasian Journal on Ageing.

[R8] Bjørkløf GH, Helvik A-S, Ibsen TL, Telenius EW, Grov EK, Eriksen S (2019). Balancing the struggle to live with dementia: a systematic meta-synthesis of coping. BMC Geriatrics.

[R9] Braun V, Clarke V (2021a). Can I use TA? Should I use TA? Should I not use TA? Comparing reflexive thematic analysis and other pattern-based qualitative analytic approaches. Counselling and Psychotherapy Research.

[R10] Braun V, Clarke V (2021b). Thematic Analysis: A Practical Guide.

[R11] Cahill S (2022). New analytical tools and frameworks to understand dementia: what can a human rights lens offer?. Ageing & Society.

[R12] Chang CYM, Baber W, Dening T, Yates J (2021). ‘He just doesn’t want to get out of the chair and do it’: the impact of apathy in people with dementia on their carers. International Journal of Environmental Research and Public Health.

[R13] Cheron EJ, Ritchie JB (1982). Leisure activities and perceived risk. Journal of Leisure Research.

[R14] Collins R, Owen S, Opdebeeck C, Ledingham K, Connell J, Quinn C, Page S, Clare L (2023). Provision of outdoor nature-based activity for older people with cognitive impairment: a scoping review from the ENLIVEN project. Health & Social Care in the Community 2023.

[R15] Connell J, Page SJ (2019). An exploratory study of creating dementia-friendly businesses in the visitor economy: evidence from the UK. Heliyon.

[R16] Connell J, Page S, Ward R, Clark A, Phillipson L (2021). Dementia and Place.

[R17] Connell J, Page SJ, Meyer D (2015). Visitor attractions and events: responding to seasonality. Tourism Management.

[R18] Connell J, Page SJ, Sheriff I, Hibbert J (2017). Business engagement in a civil society: transitioning towards a dementia-friendly visitor economy. Tourism Management.

[R19] Crawford DW, Godbey G (1987). Reconceptualizing barriers to family leisure. Leisure Sciences.

[R20] Day K, Carreon D, Stump C (2000). The therapeutic design of environments for people with dementia: a review of the empirical research. The Gerontologist.

[R21] DeJonckheere M, Vaughn LM (2019). Semi-structured interviewing in primary care research: a balance of relationship and rigour. Family Medicine and Community Health.

[R22] Disability Information Bureau (2022). Accessibility.

[R23] Duggan S, Blackman T, Martyr A, Van Schaik P (2008). The impact of early dementia on outdoor life: a ‘shrinking world’?. Dementia.

[R24] Dunn NJ, Strain LA (2001). Caregivers at risk?: changes in leisure participation. Journal of Leisure Research.

[R25] Genoe M (2010). Leisure as resistance within the context of dementia. Leisure Studies.

[R26] Genoe M, Dupuis SL (2014). The role of leisure within the dementia context. Dementia.

[R27] Gibson E, Ramsden N, Tomlinson R, Jones C (2017). Woodland wellbeing: a pilot for people with dementia. Working with Older People.

[R28] Godbey G, Crawford DW, Shen XS (2010). Assessing hierarchical leisure constraints theory after two decades. Journal of Leisure Research.

[R29] Han A, Radel J, McDowd JM, Sabata D (2016). Perspectives of people with dementia about meaningful activities: a synthesis. American Journal of Alzheimer’s Disease & Other Dementias.

[R30] Hendriks IH, van Vliet D, Gerritsen DL, Dröes R-M (2016). Nature and dementia: development of a person-centered approach. International Psychogeriatrics.

[R31] Innes A, Page SJ, Cutler C (2016). Barriers to leisure participation for people with dementia and their carers: an exploratory analysis of carer and people with dementia’s experiences. Dementia.

[R32] Jackson EL, Crawford DW, Godbey G (1993). Negotiation of leisure constraints. Leisure Sciences.

[R33] Keady JD, Campbell S, Clark A, Dowlen R, Elvish R, Jones L, Kindell J, Swarbrick C, Williams S (2022). Re-thinking and re-positioning ‘being in the moment’ within a continuum of moments: introducing a new conceptual framework for dementia studies. Ageing & Society.

[R34] Kitwood T (1997). The experience of dementia. Aging & Mental Health.

[R35] Kitwood T, Bredin K (1992). Towards a theory of dementia care: personhood and well-being. Ageing & Society.

[R36] Lazard L, McAvoy J (2020). Doing reflexivity in psychological research: what’s the point? What’s the practice?. Qualitative Research in Psychology.

[R37] Lister R (2007). Inclusive citizenship: realizing the potential. Citizenship Studies.

[R38] Low LF, Barcenilla-Wong AL, Brijnath B (2019). Including ethnic and cultural diversity in dementia research. Medical Journal of Australia.

[R39] Malterud K, Siersma V, Guassora A (2016). Sample size in qualitative interview studies: guided by information power. Qualitative Health Research.

[R40] Mapes N (2017). Think outside: positive risk-taking with people living with dementia. Working with Older People.

[R41] Marsh P, Kelly L (2018). Dignity of risk in the community: a review of and reflections on the literature. Health, Risk & Society.

[R42] Marsh P, Courtney-Pratt H, Campbell M (2018). The landscape of dementia inclusivity. Health & Place.

[R43] Martyr A, Nelis SM, Quinn C, Wu YT, Lamont RA, Henderson C, Clarke R, Hindle JV, Thom JM, Jones IR, Morris RG (2018). Living well with dementia: a systematic review and correlational meta-analysis of factors associated with quality of life, well-being and life satisfaction in people with dementia. Psychological Medicine.

[R44] Maxwell JA (2012). A Realist Approach for Qualitative Research.

[R45] Mitchell L, Burton E, Raman S, Blackman T, Jenks M, Williams K (2003). Making the outside world dementia-friendly: design issues and considerations. Environment and Planning B: Planning and Design.

[R46] Mmako NJ, Courtney-Pratt H, Marsh P (2020). Green spaces, dementia and a meaningful life in the community: a mixed studies review. Health & Place.

[R47] Morgan S, Williamson T (2014). How Can ‘Positive Risk-taking’ Help Build Dementia-friendly Communities?.

[R48] Moussouri T (2007). Implications of the social model of disability for visitor research. Visitor Studies.

[R49] Murroni V, Cavalli R, Basso A, Borella E, Meneghetti C, Melendugno A, Pazzaglia F (2021). Effectiveness of therapeutic gardens for people with dementia: a systematic review. International Journal of Environmental Research in Public Health.

[R50] Natural England (2016). Is it Nice Outside? Consulting People Living with Dementia and Their Carers About Engaging with the Natural Environment.

[R51] Nimrod G, Shrira A (2014). The paradox of leisure in later life. Journals of Gerontology: Series B.

[R52] Noone S, Jenkins N (2018). Digging for dementia: exploring the experience of community gardening from the perspectives of people with dementia. Aging & Mental Health.

[R53] Noone S, Innes A, Kelly F, Mayers A (2017). ‘The nourishing soil of the soul’: the role of horticultural therapy in promoting well-being in community-dwelling people with dementia. Dementia.

[R54] Office for National Statistics (2017). Leisure Time in the UK: 2015.

[R55] Page SJ, Connell J (2010). Leisure: An Introduction.

[R56] Page SJ, Connell J (2022). Ageing and the Visitor Economy: Global Challenges and Opportunities.

[R57] Rojek C (2003). Leisure Theory: Principles and Practice.

[R58] Scott TL, Jao YL, Tulloch K, Yates E, Kenward O, Pachana NA (2022). Well-being benefits of horticulture-based activities for community dwelling people with dementia: a systematic review. International Journal of Environmental Research in Public Health.

[R59] Shakespeare T, Zeilig H, Mittler P (2019). Rights in mind: thinking differently about dementia and disability. Dementia.

[R60] Steel EJ, Janeslätt G (2017). Drafting standards on cognitive accessibility: a global collaboration. Disability and Rehabilitation: Assistive Technology.

[R61] Sturge J, Nordin S, Sussana Patil D, Jones A, Légaré F, Elf M, Meijering L (2021). Features of the social and built environment that contribute to the well-being of people with dementia who live at home: a scoping review. Health & Place.

[R62] Thom KM, Blair SEE (1998). Risk in dementia – assessment and management: a literature review. British Journal of Occupational Therapy.

[R63] Van Schaik P, Martyr A, Blackman T, Robinson J (2008). Involving persons with dementia in the evaluation of outdoor environments. CyberPsychology & Behavior.

[R64] Visit England (2019). Dementia-friendly Tourism: A Practical Guide for Businesses.

[R65] Ward R, Rummery K, Odzakovic E, Manji K, Kullberg A, Keady J, Clark A, Campbell S (2022). Beyond the shrinking world: dementia, localisation and neighbourhood. Ageing & Society.

[R66] World Health Organization (2021). Developing a Dementia-inclusive Society: WHO Toolkit for Developing Dementia-friendly Initiatives.

[R67] Zeilig H, Tischler V, van der Byl Williams M, West J, Strohmaier S (2019). Co-creativity, well-being and agency: a case study analysis of a co-creative arts group for people with dementia. Journal of Aging Studies.

[R68] Zhao Y, Liu Y, Wang Z (2022). Effectiveness of horticultural therapy in people with dementia: a quantitative systematic review. Journal of Clinical Nursing.

[R69] Zieris P, Freund S, Kals E (2023). Nature experience and well-being: bird watching as an intervention in nursing homes to maintain cognitive resources, mobility, and biopsychosocial health. Journal of Environmental Psychology.

