# Supplementary material for: The dementia–nature–inclusivity nexus and the needs of people living with dementia
Source: Ageing Soc. Author manuscript; Available in PMC 2025 Aug 1. (PMC7617443; doi:10.1017/S0144686X24000199)
Supplement: 1 [file EMS194725-supplement-1.pdf]

Pre interview	Ask participant to bring along an artefact (e.g. winner's cup for cross-country running, a map book, a sea-shell) that speaks to a special place outdoors).
Introduction<br><br>Conversational	*Introduce yourself, share something about yourself and explain the purpose of the interview, then ask the person to tell you something about him/herself. This can be followed up by specific questions where things haven't been covered. You can always check again at the end of the interview you have everything you need.*
	Where are you currently living?
	Do you like living in [place]?
	Is that a city/town/countryside near coast?
	Are you working?
	Do you have any memory, mobility or sensory difficulties?<br>Is there anything that makes getting out and about challenging?
	Do you have a family member or friend supports you?
	What's your age?
	Gender
	Ethnicity
	Do you like going outside? (show smiley face options)
	Do you get to do things outside as much as you'd like? (show smiley face options)
**Special/favourite place (artefact based)**	Can you tell me about what you've brought to discuss today?
	What is about this place that makes it special for you and the person you support? What is the name of the person they support
	What do you do when you're there? Do you walk? Do you sit?
	How long do you spend there?
	What is it about the natural environment at X that you like?<br>Are there any sights, sounds, smells, tastes, textures that you enjoy when doing X / going there?
	Potential prompts: sand on feet, sun on skin
	How do these sensations make you feel?
	What kinds of emotions do you feel when at X/doing X?
	Is there anything about the natural environment at X that you don't like?
	Potential prompts: wet weather, terrain
	We have spoken a bit about the natural environment. It would be great to hear your opinion about the human side of this place.
	Do you go by yourself or with other people?<br>Do you find it easier to meet new people when you are there?<br>Do you find it easier to interact with others when you are there?
	Are there any practical reasons for visiting here?
	e.g. adequate paths, nice café, friendly staff
	Have you always gone to X/done X?
	Do you go to X/do X as much as you'd like to?

	Is there anything that gets in the way of you doing X?
	e.g. transport, information, terrain
	What would help you do more of x?
	When you touch this object does it bring back any memories? How does it make you feel?
**Going outdoors (beyond the garden)**	You've spoken about your favourite/special place, I'd like to ask about other outdoor places that you go to…
	Where else do you go?
	What do you do there?
	How long do you spend there?
	What is it that you like about X?
	Prompts:<br>*What is it about the natural environment that you like?*<br>*What about the human side? Meeting people?*
	How does being at X, doing X make you feel?
	Is there anything that you aren't doing at the moment that you would like to do?
	What would help you do this?
	How has the pandemic impacted on what you do?
**Partner offerings and aspirations**	Photos of partner images and activities listed in SM survey.
	Would you like to do any of these activities?
	Can you tell me why?
	Are there any that you wouldn't like to do?
	Can you tell me why?
**Things that get in the way**	Has what you do outside changed over time/since experiencing X?
	Thinking about the barriers to visiting outdoor spaces, what barriers apply to you? (prompts form SM survey)
	e.g. information, cost, transport, worried it won't be safe, I don't feel confident, I don't have the time, I'm worried it won't be accessible, worried there won't be help if I need it
	Have you adopted any strategies that help you when you go out?
	Who would you say decides where you go?
**What helps**	Can you think of a place that you would recommend visiting and why?
	How long do you spend there?

	What more could be done to make it easier or more enjoyable?
	Prompts: human and natural environment
**Closing**	Is there anything you would like to add about what we have discussed today? Do you have any questions for me?
