# Supplementary material for: The dementia–nature–inclusivity nexus and the needs of people living with dementia
Source: Ageing Soc. Author manuscript; Available in PMC 2025 Aug 1. (PMC7617443; doi:10.1017/S0144686X24000199)
Supplement: 2 [file EMS194725-supplement-2.pdf]

Pre interview	Ask participant to bring along a photo/object (e.g. winner's cup for cross-country running, a map book, a sea-shell) that speaks to a special place they visit outdoors with the person they support).
Introduction	Introduction: explain this interview will focus on a) his/her own experience, (b) his/her perception of pwd's experience, and (c) his/her experience of supporting the pwd in being outdoors.
Conversational	*Introduce yourself, share something about yourself and explain the purpose of the interview, then ask the person to tell you something about him/herself. This can be followed up by specific questions where things haven't been covered. You can always check again at the end of the interview you have everything you need.*
	Where are you currently living?
	Do you like living in [place]?
	Is that a city/town/countryside near coast?
	Tell me about who you support?
	What kind of memory, mobility or sensory difficulties does X have?
	Do you experience any of these difficulties at all?
	Are you working?
	Is there anything that makes getting out and about challenging?
	What's your age?
	Gender
	Ethnicity
	Do you like going outside?
	Do you get to do things outside as much as you'd like? (show smiley face options)
	What about the person you are supporting?<br>If they had to select one of these smiley faces in response to the question do they like going outside, which would they select?
	And what would they select for the question do they get to go outside as much as they'd like?
**Special/favourite place (artefact based)**	Can you tell me about what you've brought to discuss today?
	What is about this place that makes it special for you?
	What do you do with [name] when you're there? Do you walk? Do you sit?
	How long do you spend there?
	What is it about the natural environment that [name] likes at X?<br>Are there any sights, sounds, smells, tastes, textures that you enjoy when doing X / going there?
	Potential prompts: sand on feet, sun on skin
	How do you think these sensations make [name] feel?
	What kinds of emotions do you think [name] feels when at X/doing X?
	What do you feel when at X/ doing X with [name]
	Is there anything about the natural environment at X that [name] doesn't like**?**
	Is there anything about the natural environment at X that makes it more difficult for you to support [name]
	Potential prompts: wet weather, terrain

	We have spoken a bit about the natural environment in this place. It would be great to hear your opinion about the human side of this place.
	Do you go just the two of you or with other people?<br>Does [name] find it easier to meet new people when you are there?<br>Does [name] find it easier to interact with others?
	Do you find it easier to meet new people when you are there? Why?
	Are there any practical reasons for visiting here?
	e.g. adequate paths, nice café, friendly staff
	Have you always gone to X/done X?
	Do you and [name] go to X/do X as much as you'd like to?
	Is there anything that gets in the way of you and [name] doing X?
	e.g. transport, information, terrain
	What would help you and [name] do more of x?
	When you touch this object does it bring back any memories? How does it make you feel?
**Going outdoors (beyond the garden)**	You've spoken about your favourite/special place to go with [name], I'd like to ask about other outdoor places that you go together. First, I would like to ask you about how you think [name] feels about going outside and then how you find supporting them to go outside.
	Where else do you and [name] go?
	What do you do there?
	How long do you spend there?
	What is it that [name] likes about X?
	Prompts:<br>*What is it about the natural environment that [name] likes?*<br>*What about the human side? Meeting people?*
	How does being at X, doing X make [name] feel?
	How do you feel seeing [name] at X, doing X?
	Is there anything that [name] isn't doing at the moment that they would like to do?
	Is there anything you would like [name] to do more of?
	What would help [name] do this?
	What would help you support [name] to do this?
	How has the pandemic impacted on what you do?

**Partner offerings and aspirations**	Photos of partner images and activities listed in SM survey.
	Would [name] like to do any of these activities?
	Can you tell me why?
	Are there any that [name] wouldn't like to do?
	Can you tell me why?
	Would you like [name] to do these activities? Why?
	Are there any you would not like [name] to do? Why?
**Things that get in the way**	Has what [name] does do outside changed over time/since experiencing X?
	Thinking about the barriers to visiting outdoor spaces, what barriers apply to [name]?
	e.g. information, cost, transport, worried it won't be safe, I don't feel confident, I don't have the time, I'm worried it won't be accessible, worried there won't be help if I need it
	Has [name] adopted any strategies that help them when they go out?
	Have you adopted any strategies to help name when they go out?
	Who would you say decides where [name] goes?
**What helps**	Can you think of a place that you would recommend visiting and why?
	How long do you spend there?
	What more could be done to make it easier or more enjoyable for you in supporting [name] at this place
	Prompts: human and natural environment
**Closing**	Is there anything you would like to add about what we have discussed today? Do you have any questions for me?
